# ABCanDroid: A Cloud Integrated Android App for Noninvasive Early Breast Cancer Detection Using Transfer Learning

**DOI:** 10.3390/s22030832

**Published:** 2022-01-22

**Authors:** Deepraj Chowdhury, Anik Das, Ajoy Dey, Shreya Sarkar, Ashutosh Dhar Dwivedi, Raghava Rao Mukkamala, Lakhindar Murmu

**Affiliations:** 1 Department of Electronics and Communication, International Institute of Information Technology, Naya Raipur 493661, India; deepraj19101@iiitnr.edu.in (D.C.); lakhindar@iiitnr.edu.in (L.M.); 2 Department of Computer Science, RCC Institute of Information Technology, Kolkata 700015, India; cse2019023@rcciit.org.in; 3 Department of Electronics and Telecommunication, Jadavpur University, Kolkata 700032, India; deyajoy80@gmail.com; 4 Department of Electronics and Communication, B.P. Poddar Institute of Management and Technology, Kolkata 700052, India; shreya.ece319032@bppimt.ac.in; 5 Centre for Business Data Analytics, Department of Digitalization, Copenhagen Business School, 2000 Frederiksberg, Denmark; rrm.digi@cbs.dk

**Keywords:** breast cancer, artificial intelligence, noninvasive detection, deep learning, transfer learning

## Abstract

Many patients affected by breast cancer die every year because of improper diagnosis and treatment. In recent years, applications of deep learning algorithms in the field of breast cancer detection have proved to be quite efficient. However, the application of such techniques has a lot of scope for improvement. Major works have been done in this field, however it can be made more efficient by the use of transfer learning to get impressive results. In the proposed approach, Convolutional Neural Network (CNN) is complemented with Transfer Learning for increasing the efficiency and accuracy of early detection of breast cancer for better diagnosis. The thought process involved using a pre-trained model, which already had some weights assigned rather than building the complete model from scratch. This paper mainly focuses on ResNet101 based Transfer Learning Model paired with the ImageNet dataset. The proposed framework provided us with an accuracy of 99.58%. Extensive experiments and tuning of hyperparameters have been performed to acquire the best possible results in terms of classification. The proposed frameworks aims to be an efficient tool for all doctors and society as a whole and help the user in early detection of breast cancer.

## 1. Introduction

Among the various cancerous diseases that affect humanity all across the world, breast cancer is the second leading cause of cancer deaths after lung cancer [1]. This type of cancer, if not detected in the early stage, might lead to death, and accounts for a large number of fatalities among women [2]. Early detection and diagnosis can have high chances of successful treatment of this disease and decrease the physical and mental pain endured by patients.

There are many biomedical imaging techniques for the early detection of Breast Cancer such as, Digital Mammography, Ultrasound, and MRI based diagnoses [3]. However, most of the methods have serious radiation effects. Moreover, some of these tests do not even confirm the malignancy of the cancerous tissues. In such scenarios, Breast Biopsy is performed to confirm the same. A biopsy is a histopathological assessment of the microscopic structure of the tissues. The biopsy can be used to differentiate between normal tissue, benign, and malignant tissues [4]. The only disadvantage of using histopathological images is zooming and focusing on the required part, which is highly time-consuming and demands well-experienced pathologists. This is where Computer-Aided Diagnosis (CAD) plays its role, providing a highly accurate classification of those tissue images, thus providing a second opinion to the doctors [5].

With the approach of machine learning in the field of Breast Cancer Histopathology image analysis, the detection in the early stages of cancer has proved to be an excellent research area. Deep Learning, one of the most recent machine learning methods, has outperformed the old and conventional methods in many image analysis tasks [6]. The most common Deep Learning algorithms include Convolutional Neural Networks(CNNs). The application of convolutional neural networks for pattern recognition and feature extraction in medical imaging have proved to be quite successful [7]. Moreover, studies have shown that fine-tuning of pre-trained models using CNN as the base can achieve comparatively higher performance in a wide variety of medical imaging tasks. The knowledge from a pre-trained source can be used to improve the learning of the actual model, thereby increasing the performance, which is way better than traditional CNN techniques. CNNs are the structural representation of a stream of feature extraction stages across its layers. The knowledge obtained by training with image samples is primarily fed as the weights of the layers. However, in Transfer Learning models, one common strategy is to freeze the weights from the input layer up to a particular layer to use those feature extraction layers from the pre-trained weights [8]. Moreover, in Transfer Learning techniques, the models require less computational power and data as compared to Deep CNN models altogether, reducing training time. The only problem that arises is that transfer learning models suffer from low generalization capability and might lead to overfitting [9].

In this study, a transfer learning based approach is being implemented where a pre-trained CNN model will be used along with some fine-tuned layers to obtain the target task. This methodology has been implemented in breast cancer imaging since 2016, following the development of several pre-trained CNN models, including VGGNet, Inception, and ResNet, to solve visual classification tasks. The ImageNet image database would be used for the pre-trained weights [10]. The proposed work summarizes existing methods and identifies their performances on breast cancer detection.

### 1.1. Problem Statement

Presently, breast cancer diagnoses are performed majorly by various biomedical imaging techniques. However, these techniques involve the use of radiation, which might cause adverse health effects in the future. Thus only breast tissue biopsy can be treated as an effective method for diagnoses of the same. Moreover distinguishing between the malignant and normal tissues is also a hectic task when the number of patients is unexpectedly high. So, this paper proposes an android application for quick and reliable diagnoses of breast cancer wherein the user can easily upload an image and get results aided by deep learning.

### 1.2. Contributions of the Proposed Paper

The main contribution of ABCanDroid is:

- A Cloud integrated Android app for the detection of breast cancer from a Breast Histopathology image;
- The proposed work used a transfer learning based model, trained with 15,616 images and tested on 3904 images for the prediction of breast cancer;
- The model has accuracy and precision of 99.58% while tested on the 3904 images (Whole Slide Images);
- The proposed framework is of low cost and requires very minimal human intervention and as it is cloud integrated, so less performance load on the edge devices.

### 1.3. Article Structure

The rest of the paper is organized as follows: Section 2 as Related work. Section 3 is the Proposed Methodology, Section 4 is Experimental Results. Finally, Section 5 presents Conclusion and Future Works.

## 2. Related Works

Various Machine and Deep learning methods have been implemented in the field of health care systems (see Table 1). Most of the works implemented on Breast cancer detection are related to binary or multi-class classification using machine learning or deep learning. These works include performance report parameters such as accuracy, precision, recall, and F1 score. This section portrays some of the works related to Breast Cancer detection and classification.

In a work by Rakhlin et al. [11], a deep learning based system was proposed using pre-trained models such as VGG16, InceptionV3, and ResNet50 for the classification of images of breast tissue. The pre-trained models were used for feature extraction. This technique achieved an accuracy of 87.2% across image classification. In another work by Kwok et al. [12], four Deep Convolutional Neural Network(DCNN) architectures have been used for classification. They tried to increase the accuracy by several data augmentation techniques. Vang et al. [13] proposed an ensemble-learning based architecture for multi-class classification problems in breast cancer. Their classifier involved using logistic regression and Gradient boosting machine. These models failed to achieve the high accuracy, which is essential for any sort of medical imaging classification.

In a paper by Nawaz et al. [14], they implemented a fine-tuned AlexNet for the classification of breast cancer. They achieved an accuracy of 75.73% when they used a patch-wise dataset. Xu et al. [15] proposed a CNN based model for segmenting breast ultrasound images into four major tissues where the accuracy of their model reached over 80%. Fang et al. [16] proposed a novel method based on ultrasound images for breast cancer classification. Their segmentation algorithm received an accuracy of 88%. Again, in these above works the accuracy is well below 90%. Authors also have not provided any measure of incorporating the models in edge devices, which can be made readily available to users for easy diagnoses.

In another work by Reza et al. [17], they considered imbalanced data and worked upon them using Convolutional Neural Networks with different layers. They also used image data augmentation to create altered versions of the same image. They achieved an accuracy of about 85% in their work. Similar to this work, Ali et al. [18] proposed a neural architecture with a color constancy technique and achieved an accuracy of 93.5%. Their work also involved histogram equalization but its accuracy was not that good. Wang et al. [19] also used a similar technique of using CNNs with an added batch normalization layer. They employed five different types of models with several layers and came up with an efficient one that achieved an accuracy of 89%. The idea of using transfer learning in a similar work was adopted by Singh et al. [20], where they worked on imbalanced data from WSI dataset and used VGG-19 with different classifiers like logistic regression, random forest, and other dense layers.

In the work by Alzubaidi et al. [21], they presented a thorough study of how pre-training Transfer Learning models with different datasets can affect the performance of the model. In their study, the authors proved that pre-training models with a similar kind of data can significantly improve the model’s performance. In another work by Azizi et al. [22], they presented a novel Multi-Instance Contrastive Learning (MICLe) method, which makes use of pretrained ImageNet model followed by self-supervised learning on multiple unlabeled medical images of pathology of the same patient. Later, they fine-tuned the model with labelled medical images. This method greatly improved the accuracy of their model by 6–7% against existing models.

In the work by Ayana et al. [23], they focused on transfer learning methods applied on ultrasound breast image classification and detection using pre-trained CNN models. Moreover, their review on some of the most commonly used transfer learning techniques presents the potential of future research in this field. Khamparia et al. [24] in their work proposed a hybrid transfer learning model, mainly a fusion of MVGG and ImageNet, and achieved an accuracy of 94.3%. They implemented their model on the WSI dataset. They also employed image segmentation and 3D mammography in their work, which helped to acquire better results.

**Table 1:** Comparative study of related works for breast cancer detection.

Author	Works	Salient Features	Transfer Learning	Application on Edge Devices
Samala et al. [25]	Multi-Stage Transfer Learning used with Deep Neural Nets	Applicable for limited data and gain in performance	✓	×
Choudhary et al. [26]	Transfer Learning based on Structural Filter Pruning Approach	Applicable for point-of-care devices	✓	×
Deniz et al. [27]	Transfer Learning with deep feature extraction from pretrained models	Applicable for outperforming traditional ML models	✓	×
Ayana et al. [23]	Transfer Learning technique on Ultrasound Images	Applicable for better image processing	✓	×
Khamparia et al. [24]	Implemented Hybrid Transfer Learning	Applicable for increasing efficiency of model	✓	×
Zhang et al. [28]	Implemented combination of Transfer Learning and Recurrent Neural Net	Applicable for better result outcome	✓	×
Alzubaidi et al. [29]	Implemented DCNN based Double Transfer Learning model on histopathological images	Applicable for limited labelled data for classification and high precision	✓	×
Gatuha et al. [30]	Cloud based Android app for breast cancer detection using Naive Bayes classification	Applicable for ease of use	×	✓
ABCanDroid	Android based Transfer Learning Implementation on Histopathological Images	Applicable for ease of use and highly precise accuracy	✓	✓

In another work by Choudhary et al. [26], they performed thorough experiments using three popular pre-trained CNNs such as VGG19, ResNet34, and ResNet50. With the use of the VGG19 pruned model, they obtained an accuracy of 91.25%, outperforming initial methods on the same dataset. Sheikh et al. [31] made in their work a thorough comparison among six different classifier levels along with deep learning based algorithms. They inferred that some of their algorithms enhanced the performance of Breast Cancer classification to a large extent. Deniz et al. [27] used Fine Tuned AlexNet and VGG16 transfer learning models, achieving an accuracy of 91.3%. Both of the model features were extracted and concatenated for better results. In the above mentioned works, the authors have succeeded in achieving a moderately high accuracy, but the authors did not provide any method for making the model available for common use. These drawbacks have been addressed in this work. The work proposed in this paper is a cloud integrated transfer learning based android app. This facilities the app, being light-weight solving problems related to computation. Additionally, integrating the model with android app helps facilitate its availability to masses. These previously discussed papers have implemented their work on the same WSI Dataset and have gained good results.

Lastly, Alzubaidi et al. [29] provided a novel approach to resolve the issue of lack of data in medical imaging. The authors pre-trained DCNN model on a large number of unlabeled histopathological images of breast cancer. After fine tuning, the model was trained with small labeled dataset of breast cancer. This process enabled the authors to achieve an accuracy of 97.51%. The authors also applied novel double transfer learning, achieving an accuracy of 97.7%.

Gatuha et al. [30] provided a cloud based android application for breast cancer detection. The authors provided a data mining technique based on Naïve Bayes probabilistic classifier for breast cancer detection from images. They obtained an accuracy of 96.4%. In this paper, a Transfer Learning based model has been used, which produces significantly better results as compared to traditional machine learning algorithms.

### Critical Analysis

**Figure 1:**
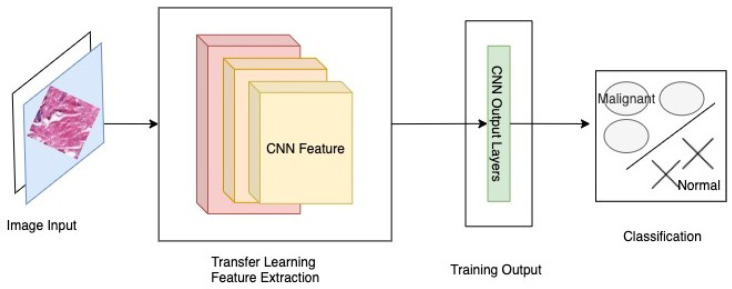
ABCanDroid model architecture.

The comparison of the proposed work with all other transfer learning and DNN based models is presented in Table 1. The research works presented above have shown quite a good performance in the detection of breast cancer when measured in terms of accuracy, but it is also necessary to implement it in a manner that would be available to patients in general. Out of all the presented works, only a few have employed the use of transfer learning. Considering the works done in terms of implementing a transfer learning based Android App, there is a requirement for a framework that gives better accuracy as well as facilitate the users with an Android Application, as shown in Figure 1.

In such a scenario, there is a need to improve the current framework benchmarks: (1) to build an association between users and the proposed framework, (2) to incorporate the best possible procedure to achieve greater accuracy, and (3) to provide an ideal experience to both patients and doctors. This proposed framework tries to improve the current scenario of existing frameworks with this research work.

## 3. Proposed Methodology

In this section, the description of the proposed approach used to achieve the objectives of the work is discussed, covering system architecture and datasets used to CNN based classification. The methodology adopted involves the use of transfer learning on the input histopathological images.

### 3.1. System Architecture

**Figure 2:**
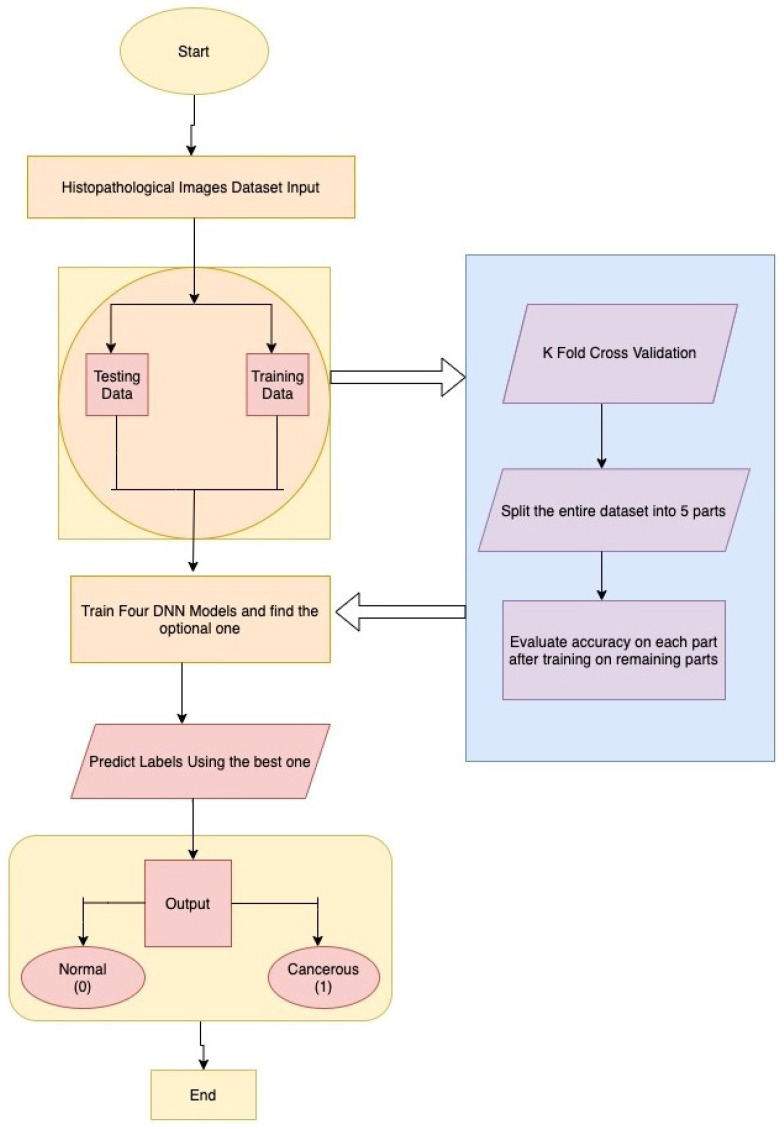
Model architecture and flowchart.

The proposed system takes an input of a Breast Histopathological Image to identify Breast Cancer. First of all, this system converts images taken from the user, from the current colour channel to the Red-Green-Blue (RGB) color channel. Furthermore, the system will consider only images that are similar to Breast Histopathological Images. First, the image is quantified through a Structural Similarity Index (SSIM) measure to check its structural similarity with a Histopathological image. Then, only the image is used for detection or classification. Figure 2 depicts the sequence of steps involving input from dataset, splitting data into testing and training and finally, after implementation, on four DNN Models and predicting the output labels. The ResNet model is pretty effective in the extraction of features and classifying images based on those features. The whole model was pushed to a serverless cloud service [32] named Heroku and then the results were fetched using an API from the Android application backend. The Android App was developed using Flutter Mobile Framework paired with the API fetching service. This was done so as to prevent the loss of accuracy when TfLite was used with Flutter.

### 3.2. Data Cleaning and Input Preprocessing

**Figure 3:**
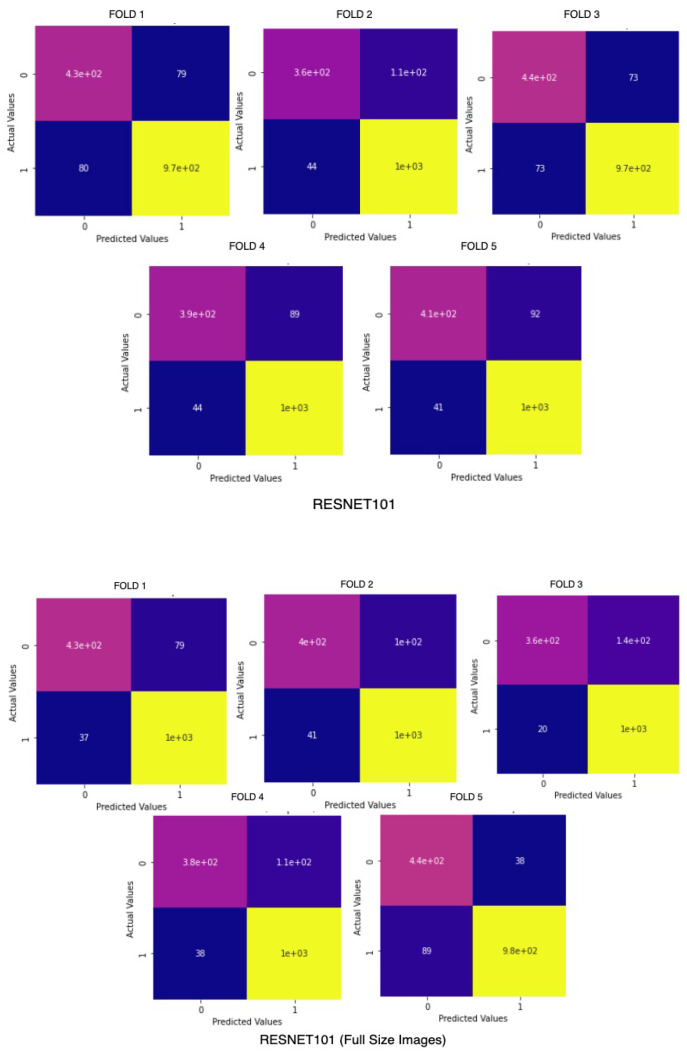
ResNet101 on BreaKHis on cropped (**above**) and original sized images (**below**).

Before the model is trained, there is a requirement of cleaning the dataset and converting it to the proper form to be fed into deep neural networks for classification. Image processing is a necessary step to achieve significant and accurate classification. The database includes images of various sizes ranging from 512 × 512 pixels to 1024 × 1024 pixels. Therefore, before passing the images for feature extraction, they have been resized to a size of 224 × 224 × 3 pixels to be ready as input to the system. The preprocessing needed for applying transfer learning on breast cancer histopathological images also involves reducing class imbalance. Moreover, the models were also trained on original sized images without any cropping or resizing to ensure there is no loss in quality or features from the image. A comparison chart of the accuracy across 5 folds of BreakHis Dataset is visualized in Figure 3.

Then, the images were labeled correspondingly using a label list that had 0 as Normal and 1 as Affected classes. Thus, the problem decreases to binary classification. The image list was then converted into a NumPy array and the dimensions were reduced within a range of 0 to 1 by dividing with 255. Then, the whole dataset was divided into Training and Testing images and labels with a ratio of 80:20, respectively.

### 3.3. Transfer Learning Approach

In medical imaging problems there is usually a scarcity of data. To overcome this problem, transfer learning has come into play and has helped to deal with small data and achieve better performance. In medical cases, especially in breast cancer imaging, different types of images are used for classification. Some of the popular types include Magnetic Resonance Imaging (MRI), Computed Tomography (CT), and Ultrasound (US). However, in this approach, the use of Histopathological images have been employed to detect and classify breast cancer images.

#### 3.3.1. Feature Extraction

**Figure 4:**
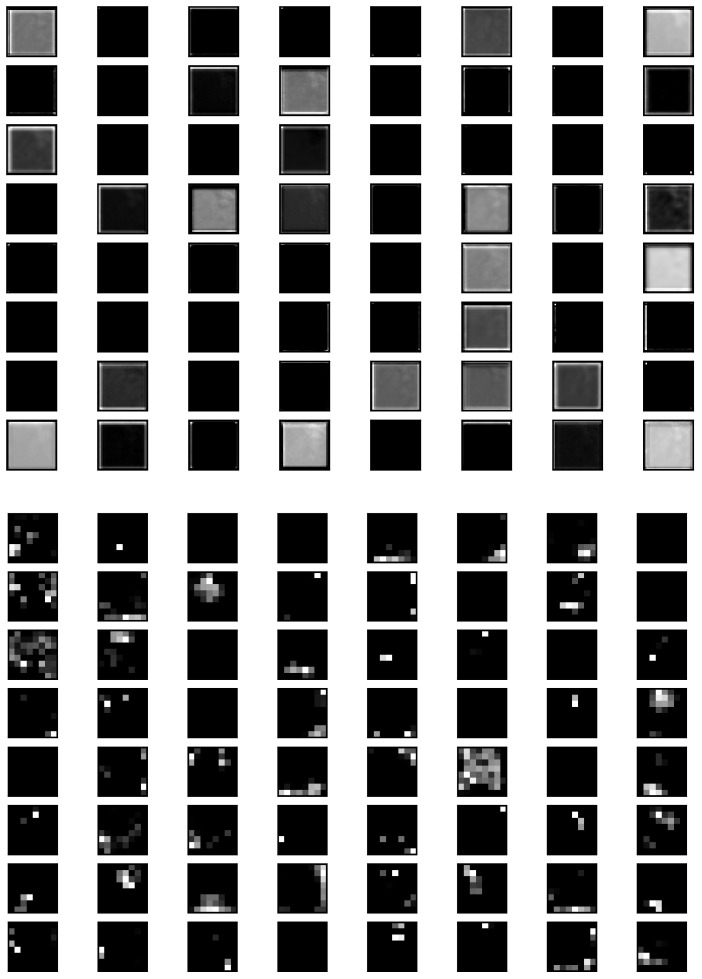
Feature map of learned features on the first two layers.

In general, there are two conventional approaches for transfer learning, namely feature extraction and fine-tuning. In the feature extraction approach, a well trained CNN model on a large dataset such as ImageNet is used to extract the features of the target domain, for example, in breast cancer imaging. The convolutional layers of the pretrained model are used as a frozen feature extractor to match with a new classification task. These features are then sent to a classifier, which is trained throughout the training process of the entire network. The feature map of the learned features obtained from a sample image when passed through the neural network architecture is visualized in Figure 4. These feature maps are from the base functional ResNet101 Layer along with the First Convolutional Layer of the neural network. The feature maps give an insight into the edges and features of the input images as it passes through various layers.

#### 3.3.2. Pretrained Model Dataset

The most common pretrained models used for transfer learning include ResNet, DensNet, Inception, and so on. Out of these, the InceptionV3 model is quite common. In this work, a comparative study of the different pre-trained models was employed to find out the best of the lot. In breast cancer imaging based on transfer learning, the ImageNet dataset is commonly used.

The ImageNet dataset is a large image database designed for image recognition tasks. It generally consists of 14 million images that have been annotated to identify pictured objects. This dataset is capable of classifying more than 20,000 categories with a particular category consisting of various images.

### 3.4. Transfer Learning CNN Models

A CNN in breast cancer image analysis is basically a feed-forward neural network. The main advantage of using CNN is its accuracy in image recognition tasks. However, it requires high computational power and huge training data. A CNN usually consists of a base input layer along with pooling and convolutional layers. Finally there is a fully connected layer, which is the output layer providing the classification results. Some of the most commonly used CNN models for transfer learning with breast cancer images are the following:

#### 3.4.1. VGG16

VGG16 was the first CNN introduced by Visual Geometry Group (VGG). VGG16 is a type of convolution neural network that consists of 13 convolution neural networks and 3 fully connected layers. This was further followed by VGG19. These architectures were based on ImageNet dataset. The main feature of VGG16 is transfer learning based CNNs used as fixed feature extractor. It is a pre-trained CNN architecture trained on a large dataset, where the last fully connected layer of this pre-trained network is removed. The remaining CNN acts as a fixed feature extractor for the new dataset.

#### 3.4.2. DenseNet

DenseNet is a more recent architecture that is used in image classification problems. It shows exceptional performances in terms of classification accuracy, despite having a fewer number of parameters. Advantages of DenseNet include parameter efficiency, in which every layer adds only a limited number of parameters. On the other side, it is more helpful because it has a higher capacity with multi-layer feature concatenation. DenseNets obtain significant improvements over the state-of-the-art on most of them, whilst requiring less memory and computation to achieve high performance.

#### 3.4.3. Xception

Xception is a convolutional neural network. It was first introduced by Google researchers. They used their idea on Xception from the Inception model because depthwise separable convolutional was better than Inception. When data is inserted to be classified, then at first it enters into the entry flow. Then, it goes through the middle flow and finally enters from the exit flow. It is a useful architecture that works on Depthwise Separable Convolution and thus makes shortcuts between convolutional blocks. Xception consists of 36 convolutional layers, and these layers take part to form the feature extraction base of networks.

#### 3.4.4. ResNet

This paper discusses three different CNN techniques—VGG16, DenseNet, and Xception, respectively. In this study, the ResNet based transfer learning technique has been implemented, which will contain ImageNet data weights downloaded from the web. Instead of building a deep learning model from scratch, a more practical approach was adopted, constructing a model using already proven models. The main advantage of ResNet model is the presence of a large number of layers. Transfer learning enables us to retrain the final layer of an existing model, resulting in a significant decrease in training time. One of the most famous models that can be used for transfer learning is ResNet.

### 3.5. The Prototype Application

**Figure 5:**
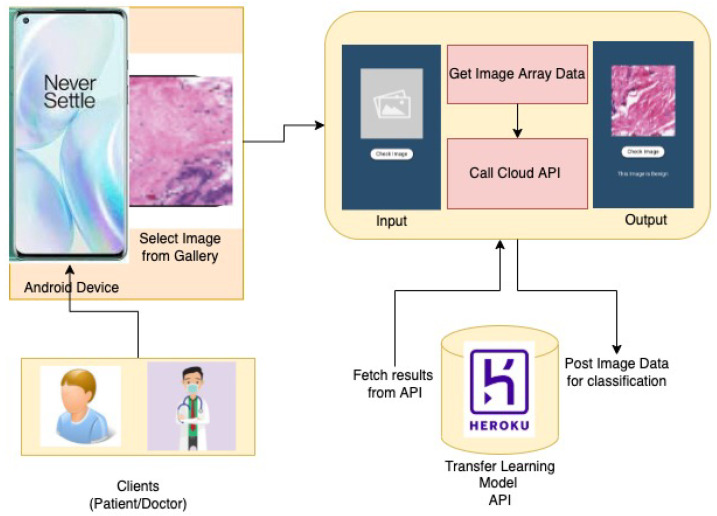
App architecture for ABCanDroid.

Following the proposed approach, a cloud integrated mobile application has been developed based on the Android operating system for the detection and classification of Breast Cancer Histopathological Images.The App Architecture Design is shown in Figure 5 The app allows users to browse and upload an image and feed it to the application. The application in turn will evaluate the image, using the model proposed and provide a classified label probability. The proposed application can be used by patients having biopsy or histopathological images obtained from clinics. In addition, doctors can utilize this application for easy diagnoses of breast cancer and also to save their time for fast and efficient diagnoses.

## 4. Experimental Details

### 4.1. Dataset Used

The experiments were conducted with the use of a publicly available histopathological image dataset based on breast cancer. The dataset is sourced from [33], and is quite a large and popular dataset. The dataset contains 198,738 IDC negative and 78,786 IDC positive whole mount slides (WSI) of Breast Cancer Specimens. Since the dataset is too large to be used as a whole, this paper makes use of 30 folders containing 19,520 images belonging to both class non-cancerous and class cancerous. This dataset was further divided into 5 folds for cross validation and the performance metrics were calculated.

**Figure 6:**
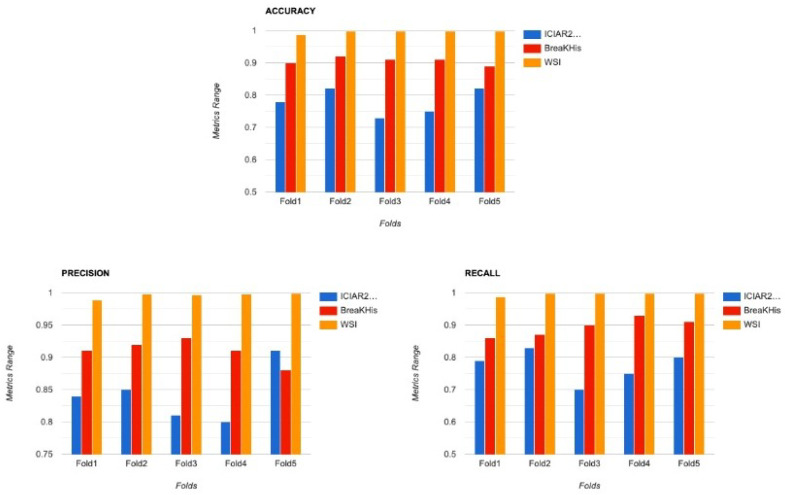
Comparison of performance metrics for WSI(O), BreaKHis(R), and ICIAR2018(B) Datasets.

Another dataset that was considered by many previous works is the BreaKHis Dataset, which is also quite popular in breast cancer classification tasks. The dataset is sourced from [34], however, it is not as huge as the previous dataset and contains only 7783 Image Samples of both benign and malignant classes. This dataset was also divided into 5 folds for cross validation. The metrics calculated were not as good as the previous one because of the smaller number of data points. A comparison chart of both the datasets has been plotted in Figure 6.

**Figure 7:**
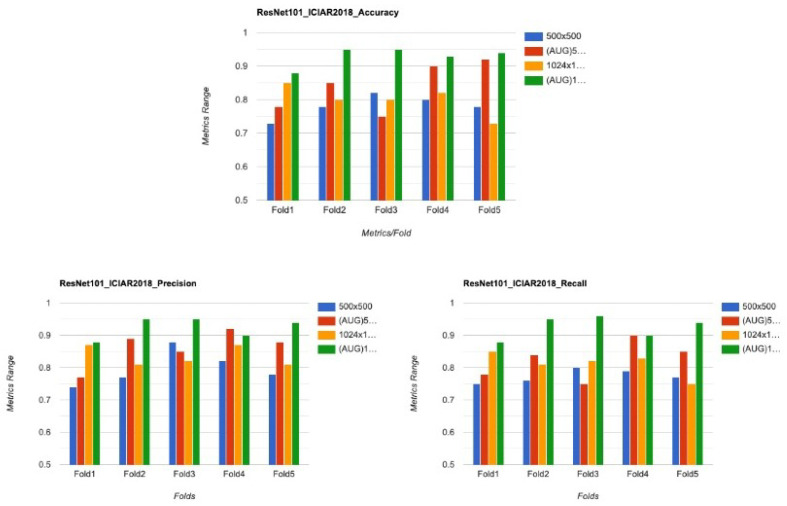
Comparison of the performance metrics ICIAR2018 for different resolutions.

The proposed work was further tested on another dataset popularly known as ICIAR2018 Dataset. The dataset is sourced from [35]. This dataset contains data from four different types of Breast Cancer. However for this study, only Benign and InSitu Invasive data points were considered. There were a total of 400 images, out of which only 200 were used for training. In order to get a good accuracy, data augmentation was done on the images. The results on this dataset are also plotted in Figure 7.

### 4.2. Experimental Setup

To analyze the performance of different models and get the best results out of them, three parameters were considered for evaluation—accuracy, sensitivity, and specificity. All the CNN models have been trained for about 15 epochs with the Adam optimizer. The time taken for model training on Google Colab is about 170 seconds/epoch for VGG16. In Xception it was taking about 154 s/epoch and for DenseNet Model it took about 157 s/epoch. Finally for ResNet Model, it was taking 214 s/epoch.

The model has been used Tensorflow 2.2.X and Python 3.X. For the training purpose, Google Colab GPU is being used. All the procedures are implemented in the Google Colab platform. The algorithm used in the experimentation is given in Algorithm 1.
**Algorithm 1** Model Input and Architecture of ABCanDroid**Require:**X:data,y:labels,z:number of images,l,b:image dimensions,f:Base Model,g:Head Model 1: **for**
i = 0 to z − 1
**do** 2:      y⇐imageLabel 3:      $image⇐image_{cvtcolor}$ 4:      $image⇐image_{resige}\left(l,b\right)$ 5:      X⇐image 6:      i++ 7: **end for**             **Model():** 8:f⇐Transfer Learning DNNs 9:g⇐Sequential()10:g·add(f)11:g·add(Dropout)12:g·add(Flatten)13:$\;g\cdot add(\mathrm{Dense}\left(\mathrm{activation}{=}^{‘}{\mathrm{softmax}}^{’}\right))$14:$\;metric⇐{(}^{‘}{\mathrm{accuracy}}^{’}{,}^{‘}{\mathrm{AUC}}^{’})$15:returnMODEL

### 4.3. Model Building

Model Building is one of the vital steps of model processing. It generally involves the construction of the base model and freezing some of the layers with pre-defined weights. The next step includes considering various popular models, training them, and finally getting their performance results. Fine-tuning of the hyper-parameters is also included while training the models to accomplish the desired accuracy. In the end, the best model out of all has been chosen and deployed to the system.

**Figure 8:**
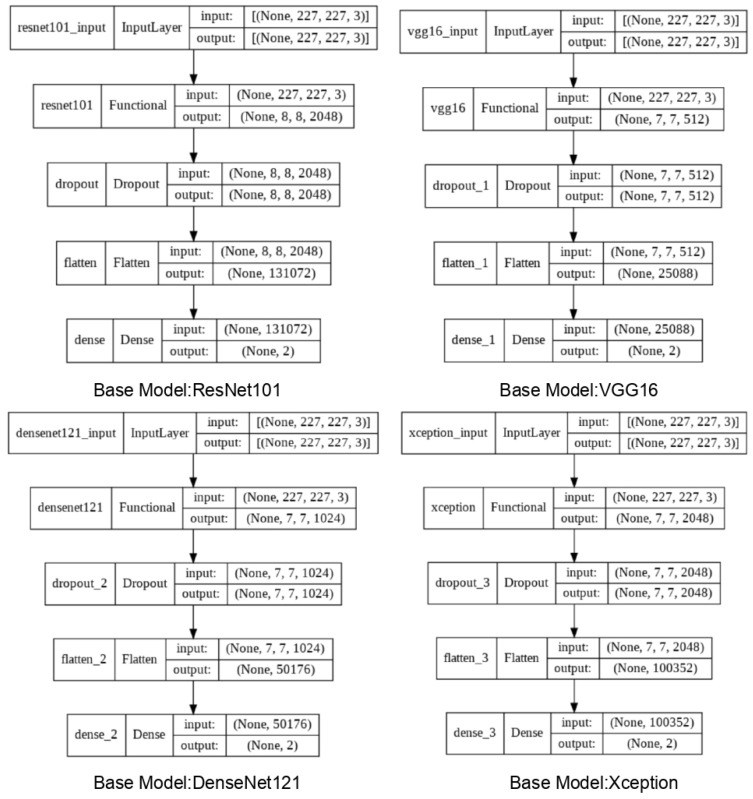
Model summary for different DNN models.

In this work, Transfer Learning has been implemented along with a Sequential Model. The summary of the models is shown in Figure 8. The sequential model enables us to create a model by adding a series of layers to the base model. It is by far the most straightforward way to build a Deep Learning Model in Keras. Each layer of the model has weights that transfer information to the layer that follows it. In this paper, four consecutive layers have been added, namely the base Pre-trained layers followed by a Dropout layer. The next communicating layer is a Flatten layer. Finally, a Softmax activated dense layer is added as the final output layer.

## 5. Results and Discussion

### 5.1. Analysis of Results

Some of the most common performance metrics include Accuracy, Sensitivity, Specificity, Precision, and so on. The formulae for the metrics are listed below. Other important metrics include True Positive (TP), True Negative (TN), False Positive (FP), and False Negative (FN).

#### 5.1.1. Evaluation Metrics

**Figure 9:**
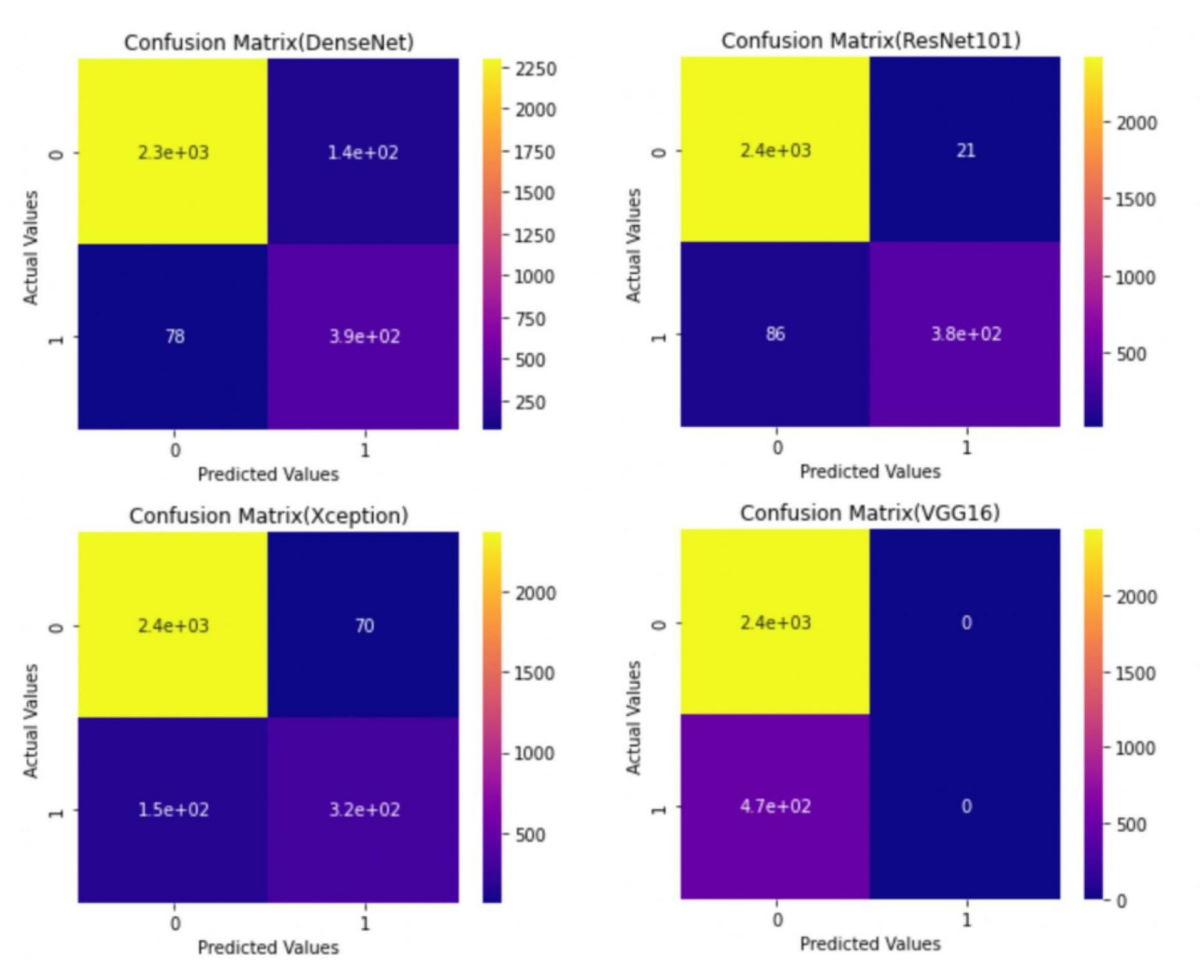
Confusion matrix for various models with 5-Fold CV (0—normal, 1—cancerous) on the WSI Dataset.

**Figure 10:**
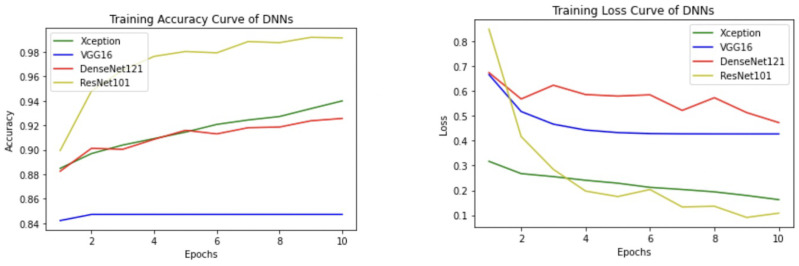
Average training accuracy and loss of 5 folds for the WSI dataset.

**Figure 11:**
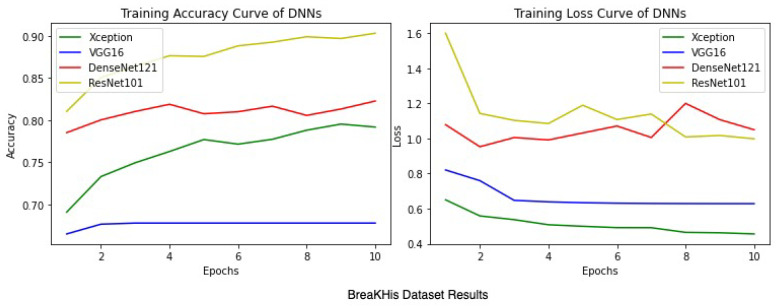
Average training accuracy and aoss of 5 folds for the BreaKHis dataset.

**Figure 12:**
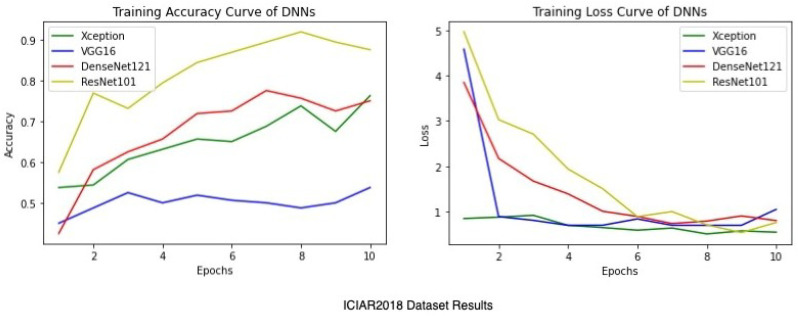
Average training accuracy and loss of 5 folds for the ICIAR2018 dataset.

**Figure 13:**
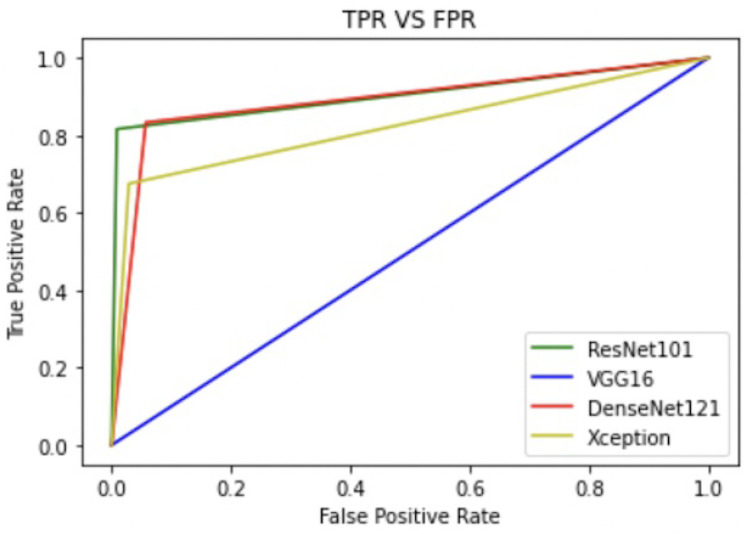
Comparison of ROC on the WSI dataset.

Apart from the metrics mentioned before, there is also a confusion matrix in Python, which is used to measure the performance of an machine learning or a deep learning algorithm. The confusion matrix of the models is visualized in Figure 9. Furthermore there are some types of graphs such as the Training Accuracy and Loss curves in Figure 10 on the WSI Dataset. The same graphs are added for BreaKHis and ICIAR2018 datasets also in Figure 11 and Figure 12. Lastly, the AUC-ROC curve, also known as the TPR-FPR curve, is also helpful for determination of the performance of the models and is visualized in Figure 13.

$$Accuracy=\frac{TP+TN}{TN+TP+FN+FP}$$

$$Sensitivity=\frac{TP}{TP+FN}$$

$$Specificity=\frac{TN}{TN+FP}$$

$$Precision=\frac{TP}{TP+FP}$$

$$F-Score=\frac{TP}{TP+(0.5)(FN+FP)}$$

$$Recall=\frac{TP}{TP+FN}$$

$$MCC=\frac{TP*TN-FP*FN}{\sqrt{\left(TP+FP)(TP+FN)(TN+FP)(TN+FN\right)}}$$

From the observed results, it can be inferred that the proposed ResNet model performs more accurately and consistently than others. This was mainly because of the increase in accuracy and lowering of loss. The increase in accuracy was due to the use of the transfer learning model over the traditional Sequential CNN model. Moreover compared to the existing systems, this work has included the model into an Android Application, which is not being implemented by most transfer learning works. All the four transfer learning frameworks achieved good results in general, but the ResNet model outperforms others when the Accuracy and AUC Scores are compared. Thus, ResNet101 was selected as the primary model for the proposed Android App.

#### 5.1.2. Cross Validation

**Figure 14:**
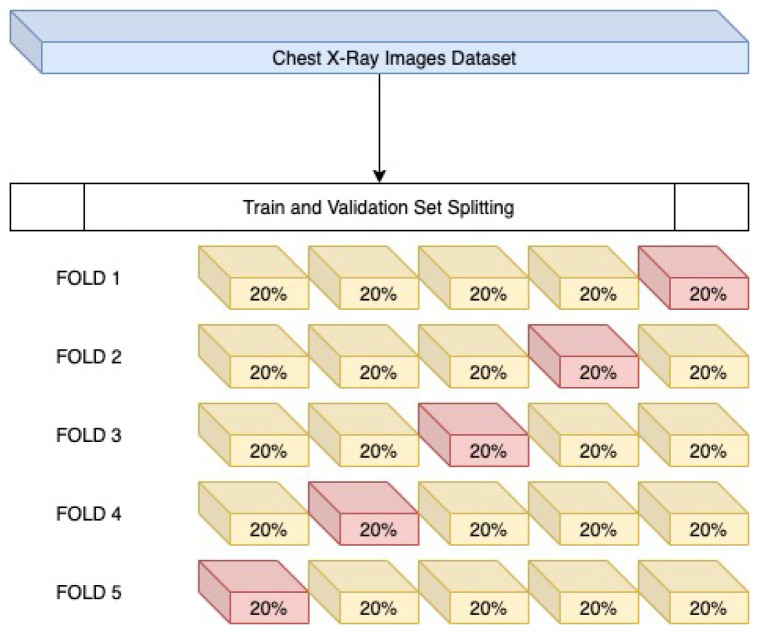
Cross validation.

To verify that the models do not get overfitted or underfitted and perform well, K-fold cross-validation has been done and was used to analyze the varying characteristics of the data. Since the data is imbalanced, performing K-fold (K = 5) validation, as shown in Figure 14, is an necessary step. It is a modification of the conventional validation and returns different fold results. The dataset is divided into five partitions and then at a given point of time one partition was used for testing the model and the rest of the parts were used for training purposes.

Table 2 and Table 3 show that the ResNet101 pretrained model outperforms all other models in terms of accuracy and precision and also in some other parameters. Table 4 and Table 5 show a comparative study of ResNet101 model performance on the different datasets used. Table 6 further shows the Cross Validation results of the ResNet101 model on WSI dataset. Table 7 shows the Cross Validation results of the ResNet101 model on BreaKHis dataset. Table 8 shows the Cross Validation results of the ResNet101 model on ICIAR2018 dataset.

## 6. Conclusions

The main goal of ABCanDroid was to create an improved breast cancer classification system that would be affordable and accessible to various healthcare systems. Considering the limitations of primitive machine learning models, the proposed study purposely used the pretrained models to extract some fine-tuned features before training on histopathological images. Several extensive experiments were performed on the four pre-trained transfer learning models, namely VGG16, DenseNet121, Xception, and finally Resnet101.

The comparative study, as shown in Table 9, indicates the superiority of transfer learning. Transfer Learning has aided the development of breast cancer diagnoses by overcoming the challenge of obtaining a large training dataset. Apart from these various preprocessing techniques, such as colour conversion, augmentation has also played a significant role in improving the performance of the model.

ABCanDroid helps users to distinguish between malignant and normal tissues by uploading a single histopathological image at a time. The proposed model has delivered an accuracy of 99.58%. In this paper, the main focus is on breast cancer detection using an android app.

## 7. Future Work

In future, the proposed work can be extended in multiple ways such as:

**IoT:** The proposed framework can be made suitable for a low power IoT device by replicating the proposed model with a lightweight model. The paper further aims at improving the potential of its architecture to be enabled in various healthcare devices. These IoT devices would allow users to curate vital data, which in turn would assist in decision making [36,37,38,39].

**Blockchain:** The proposed framework can be integrated with Blockchain to prevent data tampering. To prevent unwanted loss of patient-centric image data, a blockchain integrated Software Defined Network (SDN) can be designed. This paper aims to ensure the safety of the patient’s private data in the future [40].

**Artificial Intelligence:** Improving the performance of the deep learning model by tuning the hyperparameters. Future directions include applications of recent image processing techniques and deep learning technologies to further improve the stability and scalability of the paper [41,42,43].

**Lightweight security features:** There are no security features embedded into the ABCanDroid Framework, but in the future a cryptographic security algorithm can be implemented to provide data privacy. Lightweight encryption techniques could be implemented for secure image encryption for the healthcare industry [44,45,46,47,48]. 

## Figures and Tables

**Table 2:** Performance metrics 1 on the WSI dataset.

Model	Accuracy	Sensitivity	Specificity	AUC Score
VGG16	0.8387	0.8588	0.8188	0.6850
DenseNet121	0.9252	0.8333	0.9429	0.9471
Xception	0.9235	0.6752	0.9712	0.9260
ResNet101	0.9958	0.8162	0.9914	0.9964

**Table 3:** Performance metrics 2 on the WSI dataset.

Model	MCC	Precision	Recall	F-Score
VGG16	0.8125	0.8264	0.8387	0.7876
DenseNet121	0.7386	0.9429	0.9252	0.8847
Xception	0.7050	0.9712	0.9235	0.7966
ResNet101	0.9390	0.9958	0.9954	0.9647

**Table 4:** Performance metrics 1 of ResNet101 with the different datasets used.

Dataset	Accuracy	Sensitivity	Specificity	AUC Score
WSI	0.9958	0.8162	0.9914	0.9964
BreaKHis	0.9247	0.9300	0.8577	0.9270
ICIAR2018	0.8250	0.8636	0.8235	0.8331

**Table 5:** Performance metrics 2 of ResNet101 with the different datasets used.

Dataset	MCC	Precision	Recall	F-Score
WSI	0.9390	0.9958	0.9954	0.9647
BreaKHis	0.7877	0.9050	0.9000	0.9280
ICIAR2018	0.6872	0.8300	0.8250	0.8636

**Table 6:** The 5-fold cross validation results of the WSI dataset.

Fold	Accuracy	Precision	Recall	AUC Score
FOLD-1	0.9882	0.9882	0.9882	0.9894
FOLD-2	0.9976	0.9976	0.9976	0.9976
FOLD-3	0.9973	0.9973	0.9973	0.9976
FOLD-4	0.9976	0.9976	0.9976	0.9985
FOLD-5	0.9985	0.9985	0.9985	0.9990

**Table 7:** The 5-fold cross validation results of the BreaKHis dataset.

Fold	Accuracy	Precision	Recall	AUC Score
FOLD-1	0.8996	0.9000	0.8600	0.9115
FOLD-2	0.9152	0.9100	0.8950	0.9292
FOLD-3	0.9247	0.9050	0.9000	0.9270
FOLD-4	0.9081	0.9100	0.8900	0.9181
FOLD-5	0.8933	0.8600	0.9000	0.9115

**Table 8:** The 5-fold cross validation results of the ICIAR2018 dataset.

Fold	Accuracy	Precision	Recall	AUC Score
FOLD-1	0.7750	0.7450	0.7850	0.8212
FOLD-2	0.8250	0.8300	0.8250	0.8331
FOLD-3	0.7250	0.7050	0.7150	0.7487
FOLD-4	0.7500	0.8350	0.7500	0.7487
FOLD-5	0.8250	0.8550	0.8050	0.8206

**Table 9:** Resulting comparison of the related works.

Author	Methodology	Dataset Used	Accuracy
Singh et al. [20]	VGG-19 Transfer Learning Model	WSI	90.30%
Choudhary et al. [26]	ResNet50 Trasnfer Learning Model	WSI	92.07%
Deniz et al. [27]	Fine-Tuned AlexNet Model	WSI	93.65%
Khamparia et al. [24]	Fusion of MVGG and ImageNet	WSI	95.20%
Alzubaidi et al. [29]	DCNN Double Transfer Learning Model	WSI	98.70%
Sheikh et al. [31]	Data Augmented custom D-Net Model	WSI	98.86%
ABCanDroid	Fine-Tuned ResNet101 Model	WSI	99.58%

## Data Availability

Dataset used in this research is publicly available at https://www.kaggle.com/rangan2510/breast-cancer-histology-images (accessed on 20 December 2021).
